# Deleterious mis‐splicing of *STK11* caused by a novel single‐nucleotide substitution in the 3′ polypyrimidine tract of intron five

**DOI:** 10.1002/mgg3.1381

**Published:** 2020-06-23

**Authors:** Thorkild Terkelsen, Ole H. Larsen, Søren Vang, Uffe B. Jensen, Friedrik Wikman

**Affiliations:** ^1^ Department of Clinical Genetics Aarhus University Hospital Aarhus Denmark; ^2^ Department of Molecular Medicine Aarhus University Hospital Aarhus Denmark

**Keywords:** germline mutation, *LKB1*, Peutz–Jeghers syndrome, RNA‐Seq, splice variant, *STK11*

## Abstract

**Background:**

Pathogenic variants in *STK11*, also designated as *LKB1*, cause Peutz–Jeghers syndrome, which is a rare autosomal dominant disorder characterized by mucocutaneous pigmentation changes, polyposis, and a high risk of cancer.

**Methods:**

A male meeting the clinical diagnostic criteria for Peutz–Jeghers syndrome underwent next‐generation sequencing. To validate the predicted splicing impact of a detected *STK11* variant, we performed RNA‐Seq on mRNA extracted from patient‐derived Epstein‐Barr virus‐transformed lymphocytes treated with cycloheximide to inhibit nonsense‐mediated decay ex vivo.

**Results:**

Blood testing identified a novel single‐nucleotide substitution, NM_000455.4:c.735‐10C>A, at the end of the 3′ polypyrimidine tract of intron five in *STK11*. RNA‐Seq confirmed a predicted eight base pair insertion in the mRNA transcript. Following inhibition of nonsense‐mediated decay, the out‐of‐frame insertion was detected in 50% of all RNA‐Seq reads. This confirmed a strong, deleterious splicing impact of the variant.

**Conclusion:**

We characterized a novel likely pathogenic germline variant in intron five of *STK11* associated with Peutz–Jeghers syndrome. The study highlights RNA‐Seq as a useful supplement in hereditary cancer predisposition testing.

## Graphical abstract

We report a novel single‐nucleotide substitution c.735‐10C>A in *STK11* associated with Peutz–Jeghers syndrome. RNA‐Seq analysis confirmed a strong, deleterious splicing effect of the variant.

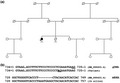

## 1 INTRODUCTION

Peutz–Jeghers syndrome (OMIM #175200) is a rare autosomal dominant disorder characterized by mucocutaneous pigmentation, gastrointestinal hamartomatous polyposis, and a high risk of various neoplasms (Jeghers, Mc, & Katz, 1949; van Lier et al., 2010). Germline pathogenic variants in the serine/threonine protein kinase 11 gene (*STK11*, also designated as *LKB1*; OMIM *602216) cause Peutz–Jeghers syndrome (Hemminki et al., 1998; Jenne et al., 1998). Traditional testing methods detect pathogenic variants in *STK11* in approximately 80% of individuals with Peutz–Jeghers syndrome (Volikos et al., 2006). Techniques focused on the transcriptome, such as whole RNA‐sequencing (RNA‐Seq), could add to the diagnostic yield by detecting deep intron variants (Abed, Gunther, Kraus, Hohenberger, & Ballhausen, 2001). We report an individual with Peutz–Jeghers syndrome due to a novel single‐nucleotide substitution c.735‐10C>A in the 3′ polypyrimidine tract of intron five in *STK11*. The study highlights RNA‐Seq as an efficient tool to assess potential splice variants.

## 2 MATERIALS AND METHODS

### 2.1 Ethical compliance

The patient gave informed consent to participate in the study. The study did not require approval from the ethics committee system in Denmark.

### 2.2 Clinical data

**Figure 1:**
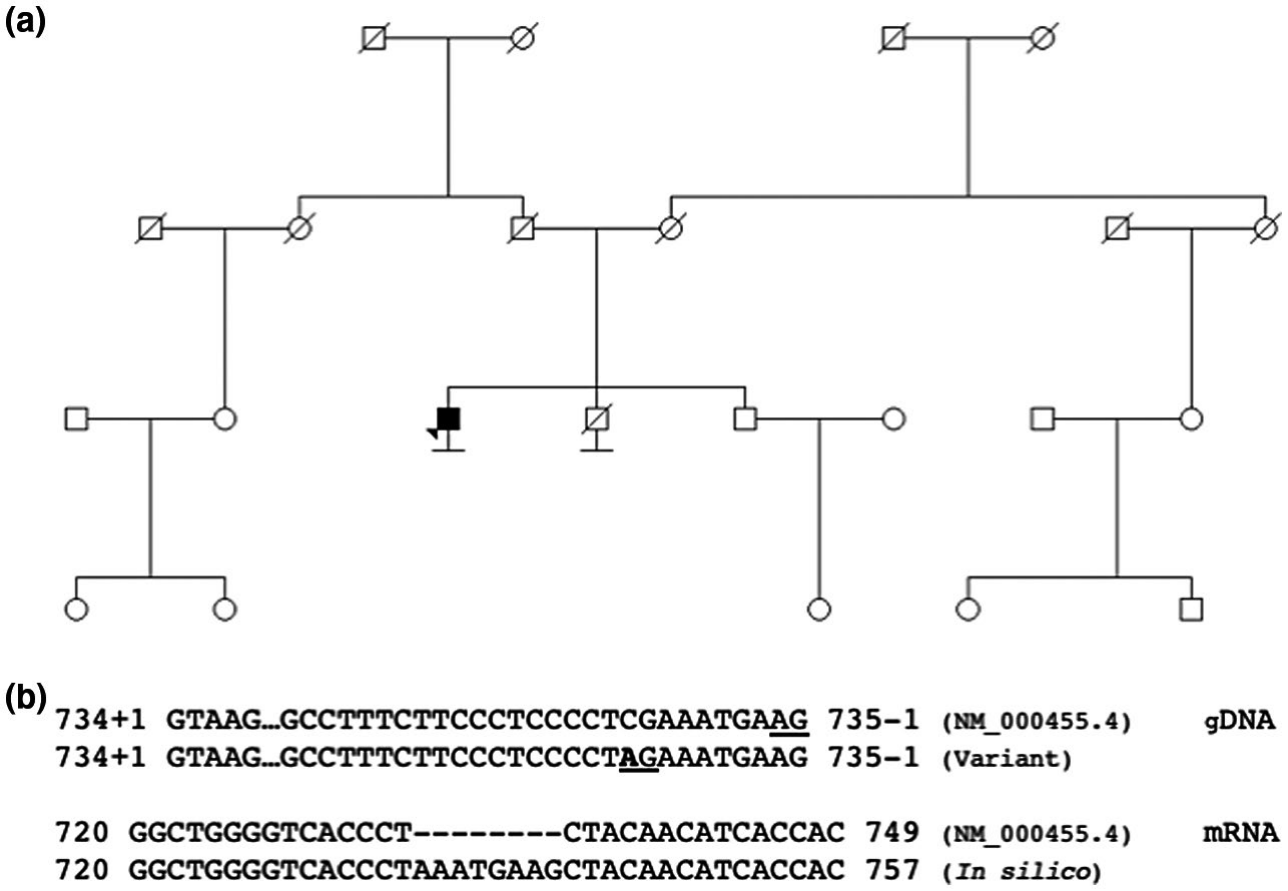
Summary of the genetic findings. (a) The pedigree of the male affected by Peutz–Jeghers syndrome and his unaffected family members. (b) The novel genomic variant in intron five of *STK11*, NG_007460.2(NM_000455.4):c.735‐10C>A, created a strong potential splice acceptor site (upper alignment) and was predicted to result in an aberrant *STK11* transcript that included the last eight bp of the intron (lower alignment)

A 60‐year‐old male underwent a screening colonoscopy in the biannual population screening program for colorectal cancer. An adenocarcinoma was identified in the sigmoid colon along with multiple polyps throughout the colon and rectum. Pathological assessment of a polyp revealed the classical Peutz–Jeghers appearance of thick smooth muscle bundles between nonneoplastic epithelial crypts. Multiple additional polyps in the upper and lower bowel demonstrated the same hamartomatous morphology, which confirmed the clinical diagnosis of Peutz–Jeghers syndrome. The patient had no family history of Peutz–Jeghers syndrome (Figure 1a).

### 2.3 DNA analysis

DNA was extracted from a blood sample of the patient and analyzed by next‐generation sequencing of a gene panel for hereditary polyposis and colorectal cancer (Table S1). All exons plus 20 base pairs (bp) of adjacent intronic sequence were analyzed. Copy number variants were identified by bioinformatic analysis of next‐generation sequencing data using ExomeDepth (Plagnol et al., 2012) and Delly2 (Rausch et al., 2012). Multiplex ligation dependent probe amplification (MLPA) was also performed on the three genes: *MLH1*, *MSH2,* and *PMS2*. Splice effects were predicted in silico using SpliceSiteFinder‐like (Zhang, 1998), MaxEntScan (Yeo & Burge, 2004), NNSPLICE (Reese, Eeckman, Kulp, & Haussler, 1997), and GeneSplicer (Pertea, Lin, & Salzberg, 2001). Population allele frequencies were extracted from the Genome Aggregation Database (gnomAD entry April 2020) (Karczewski et al., 2020) and variants were looked up in the Human Gene Mutation Database (Stenson et al., 2017) and ClinVar records (Landrum et al., 2018). The variants were classified as recommended by the American College of Medical Genetics and Genomics and the Association for Molecular Pathology (ACMG‐AMP) (Richards et al., 2015). Only variants classified as C3‐uncertain significance, C4‐likely pathogenic, or C5‐pathogenic were reported by the laboratory.

Sequence variant descriptions followed the guidelines of the Human Genome Variation Society (den Dunnen et al., 2016), and the nomenclature was checked using Mutalyzer (Wildeman, van Ophuizen, den Dunnen, & Taschner, 2008). The locus‐specific database Leiden Open Variation Database (LOVD) was used for variant submission (Fokkema et al., 2011).

### 2.4 Cell culture

Treatment of human cells with the translation elongation inhibitor cycloheximide (CHX) impairs the nonsense‐mediated decay regulatory mechanism (Carter et al., 1995), which otherwise eliminates mRNA transcripts harboring premature termination codons (Khajavi, Inoue, & Lupski, 2006). An Epstein‐Barr virus‐transformed lymphocyte culture was established from a blood sample of the patient. Immortalized lymphocytes were grown in three 75 cm^2^ culture flasks containing 19 ml culture medium added 1 ml CHX solution to final concentrations of 0, 10, or 40 µg/ml. Cells were incubated for 8 hr at 37°C, 5% CO_2_ before RNA extraction using the RNeasy Mini Kit Cat. no. 74106 (QIAGEN Nordic).

### 2.5 RNA‐Seq

Paired de‐multiplexed FASTQ files were generated using the software bcl2fastq (Illumina) and quality checked using FastQC (Andrews, 2010) and FastQ Screen (Wingett & Andrews, 2018). Adapter sequences were trimmed using TrimGalore (Krueger, 2012) and Cutadapt (Martin, 2011). Statistics were generated for the raw and trimmed FASTQ sequences for each sequence library within each sample. The trimmed reads were mapped to the human reference genome (hg19) with masked PAR1 and PAR2 regions on the Y chromosome to better estimate the expression of pseudoautosomal genes on the X chromosome. TopHat (Trapnell, Pachter, & Salzberg, 2009) was run in strand‐aware mode using the “—library‐type fr‐secondstrand” option.

To find the reads containing the predicted novel transcript that would not align to the normal RNA sequence for *STK11* (NM_000455.4), a sequence was constructed from the 10 last bases of exon 5, the 8 last bases of intron 5 (the predicted inserted sequence), and the 20 first bases of exon 6. This sequence was used as search string for agrep (Wu & Manber, 1992), allowing a maximum of 2 misaligned bp, to search the unmapped reads for reads originating from the new transcript. The findings were confirmed using the hg38 genome build and an alternative mapper STAR v2.7.2b (Dobin et al., 2013).

The RNA‐Seq reads were assumed to follow a binomial distribution between the two transcripts. Two‐sided exact binomial confidence intervals and test statistics were reported. Statistical significance was considered at the 95% confidence level.

The statistical analysis was performed in Stata v12.1 (StataCorp).

## 3 RESULTS

In the 60‐year‐old male meeting the clinical diagnostic criteria for Peutz–Jeghers syndrome, a germline variant was identified in *STK11*, NG_007460.2(NM_000455.4):c.735‐10C>A. The variant lead to a substitution of a cytosine with an adenine base at the end of the terminal polypyrimidine tract of intron five, which could potentially create a strong consensus splice acceptor site (Coolidge, Seely, & Patton, 1997). All four in silico tools predicted the variant to create a novel strong consensus splice acceptor site eight bp upstream of the natural splice acceptor site in intron five. This would lead to a splice product with an out‐of‐frame insertion of the last eight bp of intron five, r.734_735ins735‐8_735‐1, predicted to be a target of nonsense‐mediated decay due to a premature termination codon in exon 6, p.Tyr246Asnfs*44 (Figure 1b).

The variant was not observed previously in more than 120,000 healthy individuals in the Genome Aggregation Database. According to the Human Gene Mutation Database, the variant had been reported once as disease‐causing in an individual with a confirmed diagnosis of Peutz–Jeghers syndrome, yet, further data were not available about the family (Lim et al., 2004). At the same position, thymine or guanine substitutions were observed at low frequencies in the Genome Aggregation Database and not predicted to affect splicing in silico.

**Figure 2:**
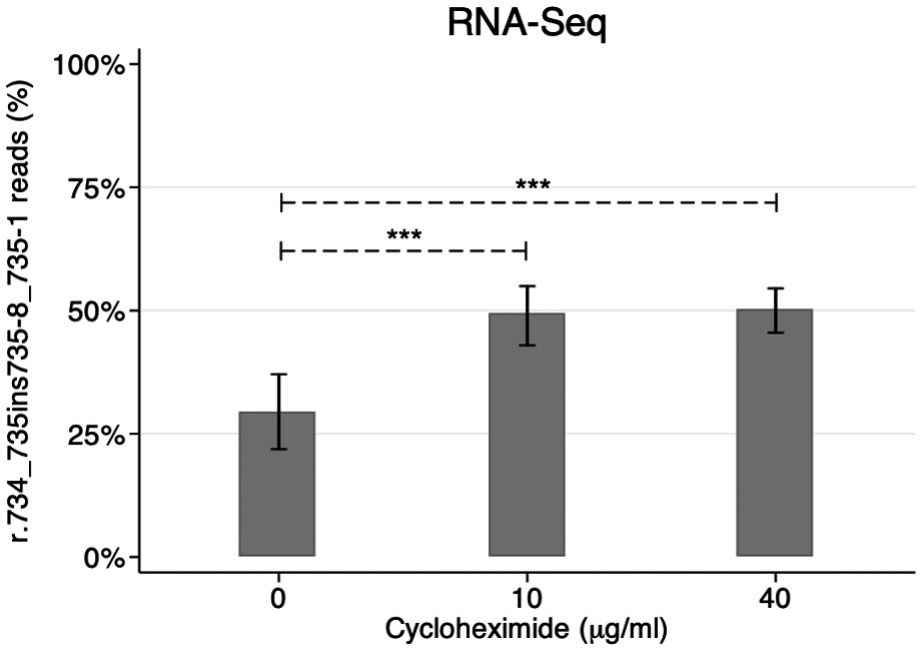
RNA‐Seq validation of the splicing impact. The figure shows the proportion of *STK11* (NM_000455.4) RNA‐Seq read counts (%) of the variant transcript with the error bars indicating the 95% confidence interval. A higher frequency of the variant transcript was observed when the nonsense‐mediated decay mechanism was inhibited with cycloheximide (*p* < .001, exact binomial test)

To investigate whether the variant affected splicing of the transcript, we performed RNA‐Seq on immortalized lymphocytes from the patient. The predicted deleterious splice product was detected at a low frequency in the absence of CHX. Yet, when the culture medium was supplemented with CHX to inhibit nonsense‐mediated decay, the RNA‐Seq read frequency of the frameshift transcript increased to 50%. This indicated a strong splicing impact of c.735‐10C>A leading to complete mis‐splicing of the transcript (Figure 2).

Interestingly, most of the aberrant transcript reads did not readily map to the reference genome but had to be identified using an agrep search string in the pool of unmapped reads (Table 1). Yet, these findings were confirmed using the hg38 genome build and the alternative mapper STAR.

**Table 1:** *STK11* RNA‐Seq read counts

Cycloheximide (µg/ml)	RefSeq^a^			r.734_735ins735‐8_735‐1^a^		
	Mapped	Unmapped	Total	Mapped	Unmapped	Total
0	98 (93%)	7 (7%)	105	3 (7%)	40 (93%)	43
10	134 (94%)	9 (6%)	143	33 (24%)	104 (76%)	137
40	242 (98%)	5 (2%)	247	17 (7%)	230 (93%)	247

^a^ GenBank reference sequence for *STK11* (NM_000455.4).

We have submitted the *STK11* sequence variant to the locus‐specific database Global Variome shared LOVD (https://databases.lovd.nl/shared/variants/0000647291).

## 4 DISCUSSION

We characterized a novel intron five variant in *STK11* associated with Peutz–Jeghers syndrome. Located between the natural splice acceptor site and a cytosine‐rich polypyrimidine tract, the variant could potentially create a strong splice acceptor site (Coolidge et al., 1997). RNA‐Seq confirmed that the variant resulted in a novel transcript with a deleterious out‐of‐frame insertion, which underwent selective degradation as a target of NMD (Khajavi et al., 2006). Peutz–Jeghers syndrome is caused by heterozygous loss of function of *STK11* (Ylikorkala et al., 1999), hence, we may assume the cytosine to adenine substitution, c.735‐10C>A, to be a causative variant. Although it might have been interesting to compare the expression of *STK11* between normal and affected tissue, immunohistochemical analysis of *STK11* expression was not part of the routine clinical work‐up for Peutz–Jeghers syndrome. Analyzing lymphoblastoid cells instead of colorectal tissue may have affected the distribution of alternatively spliced transcripts in a potential limitation of the RNA‐Seq analysis.

Since the parents were not known to be affected by Peutz–Jeghers syndrome, and because the variant was not detected in any of the relatives undergoing testing, we suspect that the variant had most likely occurred de novo. However, no tissue was available for testing from the diseased parents, which meant that we were unable to confirm this.

RNA‐Seq, as a supplement to diagnostic germline testing, has been shown to improve the outcome of hereditary cancer predisposition testing (Karam et al., 2019). Furthermore, our analysis highlights the importance to explore the unmapped RNA‐Seq reads. A low fraction of the mapped RNA‐Seq reads demonstrated mis‐splicing, yet, only the unmapped RNA‐Seq reads revealed the full impact of the splice variant.

In conclusion, this study establishes a deleterious splicing impact of c.735‐10C>A. In lack of additional clinical data to confirm its pathogenicity, we classify the variant as likely pathogenic following the guidelines of the ACMG‐AMP (Richards et al., 2015).

## CONFLICTS OF INTEREST

The authors declare no conflicts of interest.

## AUTHOR CONTRIBUTIONS

The study was conceived and designed by all authors. UBJ and TT collected the clinical data. FW, OHL, and SV lead the sequencing experiments. FW and SV performed the bioinformatic analyses. FW and TT analyzed the results and wrote the first draft of the manuscript. All authors revised the manuscript for intellectual content, gave approval of the final version to be published, and agree to be accountable for all aspects of the work in ensuring that questions related to the accuracy or integrity of any part of the work are appropriately investigated and resolved.

## Supporting information

Table S1

## Data Availability

The data that support the findings of this study are available on request from the corresponding author. The data are not publicly available due to privacy or ethical restrictions.
