# Supplementary material for: Deleterious mis‐splicing of STK11 caused by a novel single‐nucleotide substitution in the 3′ polypyrimidine tract of intron five
Source: Mol Genet Genomic Med. 2020 Jun 23;8(9):e1381. doi: 10.1002/mgg3.1381 (PMC7507455; doi:10.1002/mgg3.1381)
Supplement: Supplementary file 1 — Table S1 [file MGG3-8-e1381-s001.docx]

| **Supp. Table S1. Genes in the hereditary polyposis and colorectal cancer gene panel** | |
| --- | --- |
| Gene | RefSeq |
| *APC* | NG_008481.4 |
| *AXIN2* | NG_012142.1 |
| *BMPR1A* | NG_009362.1 |
| *EPCAM* | NG_012352.2 |
| *GREM1*^†^ | NG_033791.2^†^ |
| *MLH1* | NG_007109.2 |
| *MSH2* | NG_007110.2 |
| *MSH3* | NG_016607.2 |
| *MSH6* | NG_007111.1 |
| *MUTYH* | NG_008189.1 |
| *NTHL1* | NG_008412.1 |
| *PMS2* | NG_008466.1 |
| *POLD1* | NG_033800.1 |
| *POLE* | NG_033840.1 |
| *PTEN* | NG_007466.2 |
| *SMAD4* | NG_013013.2 |
| *STK11* | NG_007460.2 |
| † Analysis of the *GREM1*-enhancer. | |
